# An especial transition phase of hospitals: the adaptation of hospital operations to the development of COVID-19 and policy adjustments

**DOI:** 10.1186/s12199-020-00891-4

**Published:** 2020-09-21

**Authors:** Xiucheng Liu, Wei Zhuang, Xiaoyu Quan, Yeqing Zhou, Hao Qin, Chenghang Zou, Hao Zhang

**Affiliations:** 1 Department of Thoracic Surgery, Affiliated Hospital of Xuzhou Medical University, Xuzhou, 221006 Jiangsu China; 2 Thoracic Surgery Laboratory, Xuzhou Medical University, Xuzhou, 221006 Jiangsu Province China; 3 Putuo District People’s Hospital, Shanghai Key Laboratory of Signaling and Disease Research, School of Life Sciences and Technology, Tongji University, Shanghai, 200092 China; 4 Xuzhou Medical University, Xuzhou, 221006 Jiangsu Province China

**Keywords:** COVID-19, Hospital, Transition phase, New normal

## Abstract

The ongoing pandemic coronavirus disease 19 (COVID-19) remains a significant issue for global health, economics, and society. In order to balance epidemic control and economic recovery, many countries have successively announced the gradual relaxation of some lockdown restrictions. Hospitals and medical staff constitute the backbone in this war against COVID-19. In response to this serious situation, many hospitals went into emergency and impaired healthcare access to patients with conditions other than COVID-19. Therefore, gradually promoting hospital operations and functions back to the new normal is important, especially when this outbreak has been effectively controlled. In this study, we introduce existing and potential problems that could seriously affect people’s health. Additionally, we propose that an especial transition phase between the emergency and regular modes of hospitals can be well adapted to the current situation.

## Background

On March 11, 2020, the World Health Organization (WHO) declared coronavirus disease (COVID-19) to be a pandemic of international concern. To date, it has swept into over 200 countries and resulted in over 25 million cases of infection and more than 840,000 deaths. As Europe and North America have become the epicenters of the COVID-19, several countries continue to respond to the coronavirus pandemic with unprecedented rigor [1]. There is little doubt that the subsequent step after the effective control of the outbreak is to implement measures to bring the society to a new normal. Since March, these measures and efforts are being implemented across China including the Hubei province, the hardest-hit region. Considering, the enormous economic losses, and an increase in unemployment, Germany, Austria, and Italy, among other countries, announced the lifting of some lockdown restrictions in early May 2020. As important as it is to restart the economy, it is equally important to gradually drive hospital operations from the emergency to regular mode once the outbreak has been effectively controlled. Therefore, it is an ongoing challenge to balance the normalization of hospital operations and the containment protocols to keep COVID-19 in check.

### The emergency mode of public hospitals

In China, public hospitals have formed the backbone in this war against the epidemic. Right from the onset of the outbreak, local governments had immediately designated hospitals exclusively for COVID-19 [2]. These hospitals have set up temporary fever clinics and isolation sections for suspected and laboratory-confirmed cases of COVID-19 and given priority to providing medical resources, such as protective equipment and respirators, for patients with COVID-19 and the front-line medical staff. In addition, to mobilize healthcare resources and reduce the risk of exposure and cross-contamination, most hospitals have reduced the volumes of surgeries and patients visiting outpatient clinics. In London, the United Kingdom’s National Health Service has implemented several interventions in response to the pandemic, such as delaying elective operations and non-urgent radiological scans, and re-purposing clinical areas and infusion suites to form novel front-line wards [3]. These expedient measures would impair access to healthcare and emergency services for non-COVID-19-related matters to varying degrees and possibly result in the loss of innocent lives. Therefore, after the epidemic has been effectively controlled, it is imperative to take measures to re-normalize the operation of hospitals.

### Existing and potential problems

#### Increased risk of exposure and cross-contamination in hospitals

Relaxing restrictions could lead to a strong rebound in the demands of patients regarding healthcare services. In the Affiliated Hospital of Xuzhou Medical University, a large public hospital in Jiangsu Province, the number of patients visiting the outpatient clinic and surgical volume in March increased by 145.0% and 195.7%, respectively, compared to the corresponding figures in February. Hospitals are high-risk areas for infection and an excessive increase in surgical and outpatient clinic volumes could potentially lead to the gathering of crowds and disorderly management. Recently, it was reported that more than 80 patients were infected with severe acute respiratory syndrome coronavirus-2 (SARS-CoV-2), including 6 nurses and 2 doctors from a hospital outbreak in The First Affiliated Hospital of Harbin Medical University, Harbin, Heilongjiang Province. Normalizing hospital operations too early may increase the risk of exposure and cross-contamination, which may have a catastrophic effect on both the elderly patients and medical staff.

#### COVID-19–related patient fears may lead to treatment delays

Studies have shown that the elderly are most vulnerable to mortality from COVID-19 owing to weak somatic function and chronic underlying conditions, such as chronic obstructive pulmonary disease, heart disease, diabetes, and cancer [4, 5]. Undoubtedly, the evolving COVID-19 epidemic has certainly aroused fear among the general population, especially the elderly. Indeed, the fear of being infected in hospitals, “stay at home” policies, and emergency measures taken by hospitals, such as delaying elective operations and non-urgent radiological scans, have inevitably caused adverse consequences on the treatment modalities and day-to-day care of patients with chronic underlying conditions. However, this fear may last and might not disappear immediately following the easing of lockdown restrictions. For instance, after the epidemic was well controlled, some patients scheduled for surgery, including those with lung cancer and valvular heart diseases, voluntarily rescheduled their procedure at The Affiliated Hospital of Xuzhou Medical University. These delays in treatment owing to the fear instilled in the minds of patients may come at the expense of their health and lives.

#### Increased workload and psychological stress of the medical staff

During the COVID-19 outbreak, medical staff, especially those in the front-line of the epidemic, have endured enormous work-load and psychological pressure [6, 7]. Overwork, potential exposure to the virus, and a fear of infection have caused mental health problems such as tension and anxiety. Liu S et al. [8] found that the rates of depression, anxiety, insomnia, and stress symptoms among medical staff involved in epidemic prevention and control were as high as 50.7%, 44.7%, 36.1%, and 73.4%, respectively. With the gradual relaxation of policies, thousands of patients may crowd into hospitals to seek medical services. This would mean that medical staff have to invest more effort in their daily work and the management of the epidemic. However, it may be a terrible thing to push the hospital to function as before when preparation is insufficient, especially for medical workers. Therefore, there should be a transition period that offers health workers some time to overcome their existing physical and mental stress.

#### Passive decline in the quality of health care

Medical staff can continue to work on the premise that they are not infected. It is recommended to reduce unnecessary contact with patients as much as possible, especially for health staff who are not working in the front line, because asymptomatic infections are difficult to diagnose unless tested for nucleic acids [9]. Consequently, patients without COVID-19 have been indicated fewer physical examinations and less sickroom nursing; additionally, humanistic care appears to have declined during this period.

### The transitional stage between the emergency mode and regular mode of hospitals

There is no doubt that hospitals should re-enter the new normal during the post-epidemic period; however, this transition from the emergency to regular mode should be treated with caution.

- The normalization of hospitals cannot be accomplished in a single step; rather, it should be phased to ensure progressive recovery. An online pre-registration system based on identification cards may be useful for limiting the “compensatory expansion” of outpatients and decreasing surgical volumes.
- Continue to control personnel access and reduce crowd gathering. It is recommended to hire professional nurses and reduce visits from family and friends. Administrators should institute fever screening at hospital entrances and only afebrile individuals wearing face masks should be permitted into and out of hospitals. Furthermore, setting up eye-catching signs to remind people to maintain social distancing while waiting for medical examinations, treatments, and doctor’s reports could serve as a useful method.
- Designate specific hospitals and departments within hospitals to continue the treatment of patients who are suspected cases or those diagnosed with COVID-19. Other hospitals should be instructed to no longer accept patients diagnosed with COVID-19; however, fever clinics and isolation wards should be retained in these hospitals.
- Pacify the patients’ fear of COVID-19 and encourage them to actively co-operate during the treatment of primary diseases.
- Ensure that patients and staff implement hand sanitation measures at the entrance of the hospital as well as the ward.
- Nucleic acid testing especially in the respiratory, geriatric, and infectious disease departments should be implemented. In addition, nucleic acid testing should be performed on all patients prior to surgery, if conditions permit.
- More attention should be paid to the physical and mental health of all medical staff. Hospitals should implement a temporary rotation plan to allow medical staff to rest fully and adequately. Moreover, access to mental health services, such as establishing an online or offline psychological counseling platform, should be made available for front-line workers on high priority.
- Retain and improve the ability to quickly find, test, isolate, and treat symptomatic individuals or groups. Increase the ability to provide sufficient personal protective equipment and critical medical equipment. Increase the ICU capacity of hospitals to handle a dramatic surge in critical patients.

## Conclusions

During the current COVID-19 epidemic, taking “containment” measures is merely a temporary stopgap. In the near future, many countries including USA and India may lift lockdown restrictions to some extent, which may potentially lead to the unknown development of this epidemic. Hospitals, the cornerstone of the battle against COVID-19, need to adapt their operation mode to take charge of the situation and make suitable changes in implementing policies and the general social behavior to effectively combat the epidemic. We need the best of science to transform the operation mode of hospitals. The especial transitional stage between the emergency and regular modes of hospitals should be well adapted to the current situation.

## Data Availability

All data generated or analyzed during this study are included in this published article and its supplementary information files.

## Abbreviations

COVID-19: Coronavirus disease 2019
SARS-CoV-2: Severe acute respiratory syndrome coronavirus-2
WHO: The World Health Organization

## References

[1] Khosrawipour V, Lau H, Khosrawipour T, Kocbach P, Ichii H, Bania J, Mikolajczyk A (2020). Failure in initial stage containment of global COVID-19 epicenters. J Med Virol..

[2] Liu XC, Zhang DA, Sun T, Li X, Zhang H (2020). Containing COVID-19 in rural and remote areas: experiences from China. J Travel Med.

[3] El-Shakankery KH, Kefas J, Crusz SM (2020). Caring for our cancer patients in the wake of COVID-19. Br J Cancer..

[4] McMichael TM, Currie DW, Clark S, Pogosjans S, Kay M, Schwartz NG (2020). Epidemiology of COVID-19 in a long-term care facility in King County, Washington. N Engl J Med.

[5] Qiu H, Wu J, Hong L (2020). Clinical and epidemiological features of 36 children with coronavirus disease 2019 (COVID-19) in Zhejiang, China: an observational cohort study. Lancet Infect Dis.

[6] Duan L, Zhu G (2020). Psychological interventions for people affected by the COVID-19 epidemic. Lancet Psychiatry..

[7] Wu W, Zhang Y, Wang P, Zhang L, Wang GX, Lei GH, et al. Psychological stress of medical staffs during outbreak of COVID-19 and adjustment strategy. J Med ViroL. 2020. 10.1002/jmv.25914 PMC7264502 32314806

[8] Liu S, Yang L, Zhang C, Xiang YT, Liu ZC, Hu SH (2020). Online mental health services in China during the COVID-19 outbreak. Lancet Psychiatry..

[9] Zhang J, Wu S, Xu L (2020). Asymptomatic carriers of COVID-19 as a concern for disease prevention and control: more testing, more follow-up. Biosci trends..

